# Observation of the delineation of the target volume of radiotherapy in adult-type diffuse gliomas after temozolomide-based chemoradiotherapy: analysis of recurrence patterns and predictive factors

**DOI:** 10.1186/s13014-023-02203-w

**Published:** 2023-01-23

**Authors:** Hongbo Liu, Lu Zhang, Ye Tan, Yanxia Jiang, Haijun Lu

**Affiliations:** 1 Department of Radiation Oncology, The Affiliated Hospital of Qingdao University, Qingdao, China; 2 Department of Pathology, The Affiliated Hospital of Qingdao University, Qingdao, China

**Keywords:** Adult-type diffuse gliomas, Recurrence pattern, Chemoradiotherapy, Peritumoral edema

## Abstract

**Background:**

Radiation therapy is the cornerstone of treatment for adult-type diffuse gliomas, but recurrences are inevitable. Our study assessed the prognosis and recurrence pattern of different radiotherapy volumes after temozolomide-based chemoradiation in our institution.

**Methods:**

The treatment plans were classified into two groups, the plan 1 intentionally involved the entire edema area while plan 2 did not. Retrospectively investigate the differences in outcomes of 118 adult-type diffuse gliomas patients between these two treatment plans. Then, patients who underwent relapse were selected to analyze their recurrence patterns. Continuous dynamic magnetic resonance images (MRI) were collected to categorized the recurrence patterns into central, in-field, marginal, distant, and cerebrospinal fluid dissemination (CSF-d) recurrence. Finally, the clinical and molecular characteristics which influenced progression were analyzed.

**Results:**

Plan 1 (*n* = 63) showed a median progression-free survival (PFS) and overall survival (OS) of 9.5 and 26.4 months while plan 2 (*n* = 55) showed a median PFS and OS of 9.4 and 36.5 months (*p* = 0.418; *p* = 0.388). Treatment target volume had no effect on the outcome in patients with adult-type diffuse gliomas. And there was no difference in radiation toxicity (*p* = 0.388). Among the 90 relapsed patients, a total of 58 (64.4%) patients had central recurrence, 10 (11.1%) patients had in-field recurrence, 3 (3.3%) patients had marginal recurrence, 11 (12.2.%) patients had distant recurrence, and 8 (8.9%) patients had CSF-d recurrence. By treatment plans, the recurrence patterns were similar and there was no significant difference in survival. Reclassifying the progression pattern into local and non-local groups, we observed that oligodendroglioma (*n* = 10) all relapsed in local and no difference in PFS and OS between the two groups (*p* > 0.05). Multivariable analysis showed that subventricular zone (SVZ) involvement was the independent risk factor for non-local recurrence in patients with GBM (*p* < 0.05).

**Conclusion:**

In our study, deliberately including or not the entire edema had no impact on prognosis and recurrence. Patients with varied recurrence patterns had diverse clinical and genetic features.

## Introduction

Gliomas are derived from neuroglia in the brain and spine, representing over 80% of malignant central nervous system (CNS) tumors [1]. As the understanding of the molecular underpinnings of CNS tumors, the 2021 World Health Organization (WHO) classification reorganizes gliomas into adult-type diffuse gliomas, paediatric-type diffuse low-grade and high-grade gliomas, circumscribed astrocytic gliomas, and ependymal tumors [2, 3]. Adult-type diffuse gliomas are the most common pathological type of gliomas, and according to the 2021 update, they can be divided into three main disease groups based on their isocitrate dehydrogenase (IDH1/2) mutation status and 1p/19q codeletion status: IDH‐mutant, 1p/19q codeleted oligodendroglioma; IDH‐mutant, non‐codeleted astrocytoma; and IDH‐wildtype glioblastoma (GBM) [3]. Despite the important advancement achieved in surgery, radiotherapy (RT), neuro-imaging, targeted therapy, and chemotherapy for gliomas, the long-term outcome of remains poor [4, 5]. It is well documented that advanced age, poor performance status, and an insufficient amount of resection are all negative prognostic factors [6, 7].

Hundreds of current clinical trials attempt to develop new drugs for adult-type diffuse gliomas, but RT remains one of the few viable therapy options to improve both local control and survival. Whole brain radiation therapy (WBRT) is the first treatment modality and then, the target has been gradually lowered as radiation technology has advanced, yet patients' prognoses have not been shortened. Tumor edge definition is an essential component for radiotherapy. Many studies indicated that treatment failures for adult-type diffuse gliomas were more likely within 2–3 cm of the primary tumor [8, 9]. In line with this findings, Radiation Therapy Oncology Group (RTOG) [10] and European Organization for Research and Treatment of Cancer (EORTC) [11] have published two widely used radiotherapy protocols for adult-type diffuse gliomas. There is no consensus on the target delineation and the major debate lies in whether the global edema should be intentionally included. The theoretical basis for including peritumoral edema is that adult-type diffuse gliomas are so aggressive that the edema may be also an infiltrative area and patients with major edema have shorter overall survival (OS) compared to patients with minor edema [12, 13]. Choi et al. discovered that include peritumoral edema in the radiotherapy volume can reduce marginal failures after TMZ-based chemoradiation while not increasing pseudoprogression/radiation necrosiss [14]. Meanwhile, the theoretical rationale for excluding edema is that recurrence patterns are similar regardless of including edema [15, 16]. Meanwhile, the target volume, which includes the edema area, raises the radiation volume of normal brain tissue and increases the risk of brain injury [15].

To better address the delineation of the target in patients with adult-type diffuse gliomas, we retrospectively analyzed our institution's radiotherapy planning between 2017 and 2020. We found two consultants in our institution had different understandings of peritumoral edema. One of them believes that there may be tumor cells in the edema and it should be fully included in the irradiation range. The other consultant believes that a small range of targets is more suitable and has no effect on prognosis. So, we collected a total of 118 cases of adult-type diffuse gliomas were treated by these two consultants. The recurrence patterns and outcomes of the two ways were analyzed to explore whether the peritumoral edema had an effect on the patient's progression. In addition, we sought clinical features and biomarkers of preclinical pathological that could serve as early predictors for recurrence patterns.

## Methods

### Patients and cases

All patients who received definitive RT and chemotherapy for adult-type diffuse gliomas in the Affiliated Hospital of Medical College Qingdao University from 2017 to 2020 were reviewed. The inclusion criteria were: (1) age ≥ 18, (2) surgically resected and pathologically confirmed adult-type diffuse gliomas according to the 2021 WHO classification, (3) definitive chemotherapy and RT were received, and (4) the dynamic cranial MRI follow-up data can be found.

A total of 118 patients were included in the study. All treatment plans and margins were reviewed in the Varian Eclipse radiotherapy planning system. The electronic medical record (EMR, His) system was used to retrieve patient clinical, imaging, and outcome data, and telephone interviews were used to supplement deficient follow-up data. The subventricular zone (SVZ) involvement was considered tumors with enhanced lesions touched the lining of the lateral ventricle [17]. Cerebral midline involvement was regarded as the midline structures of the brain shift due to tumor or edema compression. Pathological findings were reviewed to find molecular markers such as 1p19q polysomy, IDH1, O-6 methylguanine-DNA-methyltransferase (MGMT), telomerase reverse transcriptase (TERT), ki-67 index, hematopoietic progenitor cell antigen CD34, protein 53 (P53), alpha-thalassemia/mental retardation, X-linked gene (ATRX), and the B-Raf gene mutation (BRAF V600E). All cases were reevaluated and reclassified according to the guidelines of the 2021 WHO classification of tumors of the CNS. This study was approved by our Institutional Review Committee. Prior to analysis, the patient records/information were anonymised and de-identified, and informed consent was not acquired from each participant.

### Treatment

Firstly, all the patients received a safe resection. Gross total resection (GTR) was defined as full excision of the enhanced and unenhanced masses with no visible remaining tumor. Subtotal resection (STR) was characterized as no more than 10% remaining lesion on postoperative imaging. The following treatment was RT (60 Gy, delivered in 2 Gy per fraction, Monday to Friday) for 6 weeks. As shown in Table 1, the plan 1 as follows: Gross tumor volume (GTV) was the resection cavity, postoperative enhanced area on contrast-enhanced T1-weighted magnetic resonance images (MRI), and abnormal T2-weighted and fluid-attenuated inversion recovery (FLAIR) area. Clinical target volume (CTV) was expanded from GTV by 2 cm. Planning target volume (PTV) was defined as CTV plus 3–5 mm margin. Our plan 2 was similar to the EORTC protocol as follows: GTV includes the T1-weighted MRI enhancement area and resection cavity but didn’t intentionally include the peritumoral edema area. CTV was obtained by GTV extended 2 cm. PTV was defined as CTV plus 3–5 mm margin according to each center. For intensity-modulated radiotherapy (IMRT) planning, the dose prescribed for PTV was 60 Gy in 30 fractions for both subgroups. The margins were reduced according to the barriers to tumor growth such as dura and bone are considered the and margin and eve were standardized to ensure that at least 95% of PTV got the prescribed dose.

**Table 1:** Comparison of the target definition and delineation principles in our study

Group	Plan 1	Plan 2
Phases	60 Gy	60 Gy
GTV	T1 + cavity (post-op) + T2 FLAIR	T1 + cavity (post-op)
CTV	GTV + 2 cm	GTV + 2 cm
PTV	CTV + (3–5) mm	CTV + (3–5) mm

*GTV* Gross tumor volume, *CTV* Clinical target volumes, *PTV* Planning target volume, *post-op* Postoperative, *FLAIR* Fluid Attenuated Inversion Recovery Image

Concurrent chemotherapy with TMZ should be administered daily at a dose of 75 mg/m^2^ during RT. After RT, adjuvant chemotherapy (TMZ 150–200 mg/m^2^ every 28 days) was scheduled to be performed a minimum of 6 cycles with TMZ can remain applied if the patient's physical condition permitted. The dose was reduced or chemotherapy was suspended when the patient's physical condition deteriorated and the disease advanced.

### Follow-up and identification of recurrence pattern

Subsequently, the participants after RT underwent regular follow-up visits (every 3 months) until December 31, 2021. The date of imaging following RT demonstrating radiographic progression confirmed by the attending radiation oncologist was defined as the date of the first progression. If the death occurred from suspected progression without imaging confirmation, the date of death was used as the date of tumor progression. The follow-up time was calculated from the surgery and the endpoint was the date of event occurred or the date of the last follow-up. Pseudoprogression usually occurs within 2–6 months after the end of concurrent radiochemotherapy and gradually decreases or even disappears after a few months [18]. Radiation necrosis (RN) and tumor recurrence are difficult to distinguish. The images of RN typically have increased T1 contrast enhancement, described as “Swiss-cheese” or “soap-bubble”, in the radiated area with central hypointensity and increasing edema [19]. The recurrence was identified when met the four conditions: (1) according to the RANO criteria [20] to analyze the postoperative dynamic follow-up of craniocerebral enhanced MRI; (2) multiple imaging confirmed, such as perfusion imaging (PWI), magnetic resonance spectroscopy (MRS), diffusion-weighted imaging (DWI), Arterial spin labeling (ASL) and other examination methods combined with clinical symptoms confirmed relapse; (3) pathological confirmation of reoperation after disease progression and (4) diagnoses determined through multidisciplinary consensus conferences.

**Fig. 1:**
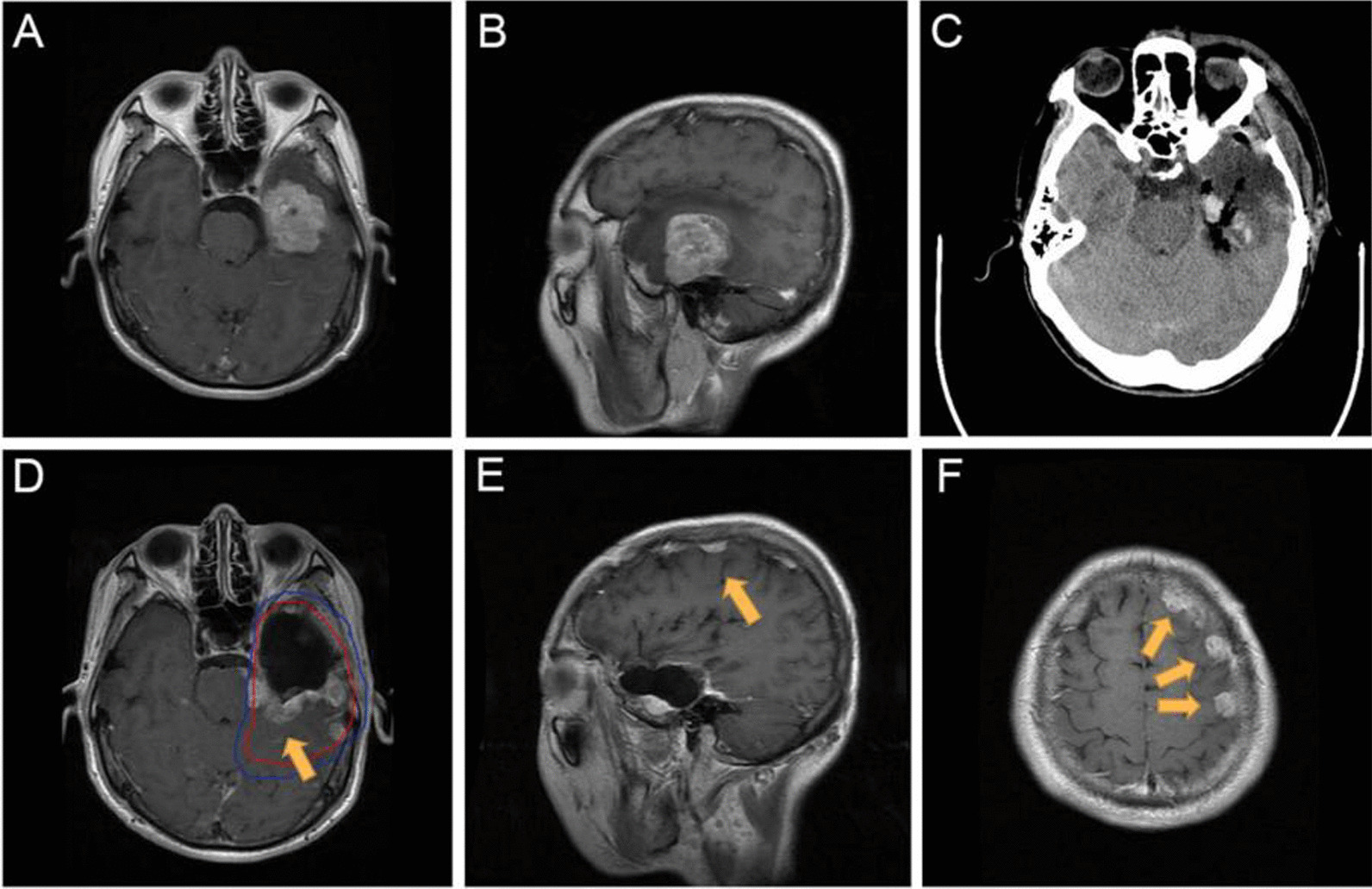
MRI scans from a patient who developed representative dissemination recurrence. **A** Preoperative axial enhanced T1-weighted images and **B** preoperative sagittal enhanced T1-weighted images showed that the tumor was in the left frontotemporal lobe. **C** CT images 1 day after operation. **D**–**F** Enhanced T1-weighted images follow-up 6 months after resection showed a new in-field lesion and multiple meningeal metastasis (yellow arrows). The blue and red lines represent the isodose lines of PTV, CTV, respectively. *MRI* Magnetic resonance imaging

The contrast-enhanced T1-weighted images of the first follow-up and tumor recurrence were imported into the Varian Eclipse radiotherapy planning system and fused with the original simcomputed tomography (CT) images. The target of the recurrent tumor was delineated, and the volume of the relapsed tumor was determined based on CT and MRI fusion images. All volumes were analyzed by reviewers blinded to the patients’ outcomes to reduce the measurement bias. Recurrence patterns were classified as follows [21]: central recurrence, in-field recurrence, marginal recurrence, and distant recurrence, where more than 95%, 80–95%, 20–80%, and lower than 20% of the recurrence volume overlaps within 60 Gy IDL, respectively. Recurrence near the ventricle or cerebral cortex may be cerebrospinal fluid dissemination (CSF-d) and is separated from distant recurrence [22]. Figure 1 illustrates the typical CSF-d images in patient with GBM.

### Statistical analysis

The summary of data was presented as the mean ± SD for parametric variables and the percentage for categorical variables. Statistical testing was performed using IBM SPSS 26.0 (IBM Corporation, Armonk, NY, USA) and R software 4.1.1 (http://www.r-project.org). Associations between RT plans and patient characteristics were calculated using the chi-square test or Fisher’s exact test for small sample sizes where appropriate and t-test for continuity variables. Survival probabilities were estimated and plotted using the Kaplan–Meier method. Chi-squared analysis was performed to determine differences in patterns of failure between subgroups. All testing is performed on the univariate level and then, the variables with *p* ≤ 0.1 in univariate analysis and clinical relevance were entered into the multivariate logistic regression analyses. Multivariate logistic regression analyses were constructed to estimate the odds ratio (OR) for outcomes with categorical variables. All statistical testing was two-sided and the p value of less than 0.05 was considered significant in this study.

## Results

### Patient characteristics

According to the criteria, a total of 118 patients were enrolled and retrospectively analyzed in the present study, including 83 (70.3%) males and 35 (29.7%) females, 76 (64.4%) GBM, 32 (27.1%) astrocytoma and 10 (8.5%) oligodendroglioma with a mean age of 52.3 years. The clinical, radiological, and molecular data of our two cohorts were shown in Table 2.

**Table 2:** Baseline characteristics of patients overall in the different target volume groups

Characteristics	Plan 1(*N* = 63)	Plan 2(*N* = 55)	Total(*N* = 118)	*P*-value
Age(years), *n* (%)				
Mean (SD)	51.9 (13.8)	52.7 (12.8)	52.3 (13.3)	
≤ 50	25 (39.7)	20 (36.7)	45 (38.1)	0.711
> 50	38 (60.3)	35 (63.6)	73 (61.9)	
Gender, *n* (%)				0.278
Female	16 (25.4)	19 (34.5)	35 (29.7)	
Male	47 (74.6)	36 (65.5)	83 (70.3)	
BMI, *n* (%)				
mean (SD)	24.5 (3.3)	25.0 (3.2)	24.7 (3.3)	
> 24	31 (49.2)	34 (61.8)	65 (55.1)	0.169
≤ 24	32 (50.8)	21 (38.2)	53 (44.9)	
Symptom of onset, *n* (%)				0.744
Consciousness disorders	4 (6.3)	3 (5.5)	7 (5.9)	
Dizziness	5 (7.9)	4 (7.3)	9 (7.6)	
Dysfunction	7 (11.1)	11 (20.0)	18 (15.3)	
Epilepsy	5 (7.9)	2 (3.6)	7 (5.9)	
Headache	30 (47.6)	27 (49.1)	57 (48.3)	
Limb weakness	12 (19.0)	8 (14.5)	20 (17.0)	
Extent of resection, *n* (%)				0.572
GTR	55 (87.3)	46 (83.6)	101 (85.6)	
STR	8 (12.7)	9 (16.4)	17 (14.4)	
Pathological diagnosis with WHO 2021, *n* (%)				0.958
Astrocytoma	17 (27.0)	15 (27.3)	32 (27.1)	
Oligodendroglioma	6 (9.5)	4 (7.3)	10 (8.5)	
GBM	40 (63.5)	36 (65.5)	76 (64.4)	
Laterality, *n* (%)				0.414
Left	34 (54.0)	23 (41.8)	57 (48.3)	
Right	24 (38.1)	27 (49.1)	51 (43.2)	
Bilateral	5 (7.9)	5 (9.1)	10 (8.5)	
Tumor location, *n* (%)				0.962
Frontal	20 (31.7)	19 (34.5)	39 (33.1)	
Occipital	2 (3.2)	1 (1.8)	3 (2.5)	
Parietal	4 (6.3)	5 (9.1)	9 (7.6)	
Temporal	20 (31.7)	15 (27.3)	35 (29.7)	
Cerebellar	2 (3.2)	1 (1.8)	3 (2.5)	
Multiple	14 (22.2)	12 (21.8)	26 (22.0)	
Ventricle	1 (1.6)	2 (3.6)	3 (2.5)	
SVZ involvement, *n* (%)				0.814
No	33 (52.4)	30 (54.5)	63 (53.4)	
Yes	30 (47.6)	25 (45.5)	55 (46.6)	
Cerebral midline involvement, *n* (%)				0.509
No	29 (46.0)	22 (40.0)	51 (43.2)	
Yes	34 (54.0)	33 (60.0)	67 (56.8)	
Brain cortex involvement, *n* (%)				0.413
No	39 (61.9)	38 (69.1)	77 (65.3)	
Yes	24 (38.1)	17 (30.9)	41 (34.7)	
MGMT promoter, *n* (%)				0.217
Unmethylated	12 (19.0)	10 (18.2)	22 (18.6)	
Methylated	20 (31.7)	8 (14.5)	28 (23.7)	
Unknown	31 (49.2)	37 (67.3)	68 (57.6)	
TERT promoter, *n* (%)				1.000
Wild type	11 (17.5)	7 (12.7)	18 (15.3)	
Mutated	18 (28.6)	10 (18.2)	28 (23.7)	
Unknown	34 (53.9)	38 (69.1)	72 (61.0)	
Ki-67 index (%), *n* (%)				
Mean (SD)	32 (19)	35 (17)	0.33 (0.18)	
≤ 20	27 (42.9)	17 (30.9)	44 (37.3)	0.181
> 20	36 (57.1)	38 (69.1)	74 (62.7)	
ATRX, *n* (%)				0.275
Wild type	14 (22.2)	14 (25.5)	28 (23.7)	
Mutated	41 (65.1)	25 (45.5)	66 (55.9)	
Unknown	8 (12.7)	16 (29.0)	24 (20.3)	
BRAF, *n* (%)				0.961
Wild type	54 (85.7)	30 (54.6)	84 (71.8)	
Mutated	2 (3.3)	2 (3.6)	4 (3.4)	
Unknown	6 (9.5)	23 (41.8)	29 (24.8)	
P53 expression, *n* (%)				**0.024**
Wild type	11 (17.5)	17 (30.9)	28 (23.7)	
Mutated	45 (71.4)	25 (45.5)	70 (59.3)	
Unknown	7 (11.1)	13 (23.6)	20 (17.0)	
CD34, *n* (%)				**0.001**
Wild type	29 (46.0)	10 (18.2)	39 (33.1)	
Mutated	25 (39.7)	36 (65.5)	61 (51.7)	
Unknown	9 (14.3)	9 (16.3)	18 (15.2)	
Recurrence, *n* (%)				1.000
Recurrence	48 (76.2)	42 (76.4)	90 (76.3)	
No recurrence	15 (23.8)	13 (23.6)	28 (23.7)	
Time to radiation(*d*), ≤ 42d (*n*%)				0.655
Astrocytoma	8 (47.1)	8 (53.3)	16 (50.0)	
Oligodendroglioma	3 (50.0)	1 (25.0)	4 (40.0)	
GBM	24 (60.0)	20 (55.6)	44 (57.9)	
Pseudoprogression/RN, *n* (%)				0.388
Yes	13 (20.6)	8 (14.5)	21 (17.8)	
No	50 (79.4)	47 (85.5)	97 (82.2)	

*GTR* Gross total resection, *STR* Subtotal resection, *GBM* Glioblastomas; *MGMT* O6-methylguanine-DNA-methyltransferase gene, *TERT* Telomerase reverse transcriptase, *SVZ* Subventricular zone, *BRAF* v-raf murine sarcoma viral oncogene homolog B1, *p53* protein 53, *CD34* Hematopoietic progenitor cell antigen CD34; *RN*  Radiation necrosis. In bold: *p* value less than 0.05

### Survival

**Fig. 2:**
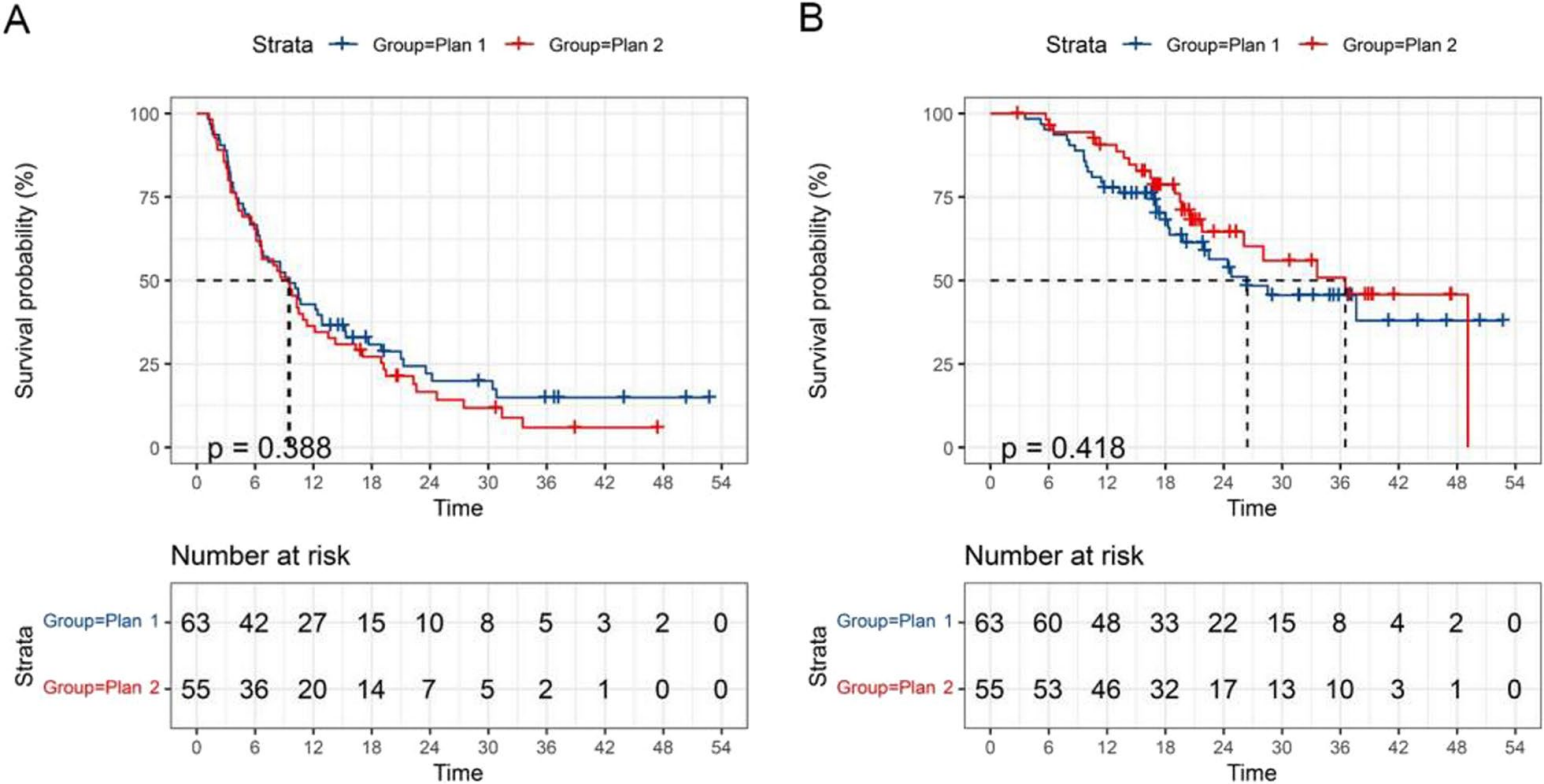
PFS curve **A** and OS curve **B** of 118 adult-type diffuse gliomas patients by different target volumes. *PFS* Progression-free survival, *OS* Overall survival

The whole cohort median follow-up was 26.4 months (range: 2.9–52.8 months). Median PFS and OS for the entire group were 9.4 and 33.6 months, respectively. As shown in Fig. 2, treatment volume did not affect outcome within the adult-type diffuse gliomas group with the plan 1 (*n* = 63) showing a median PFS and OS of 9.5 and 26.4 months and the plan 2 (*n* = 55) showing a median PFS and OS of 9.4 and 36.5 months (*p* = 0.388; *p* = 0.418).

**Fig. 3:**
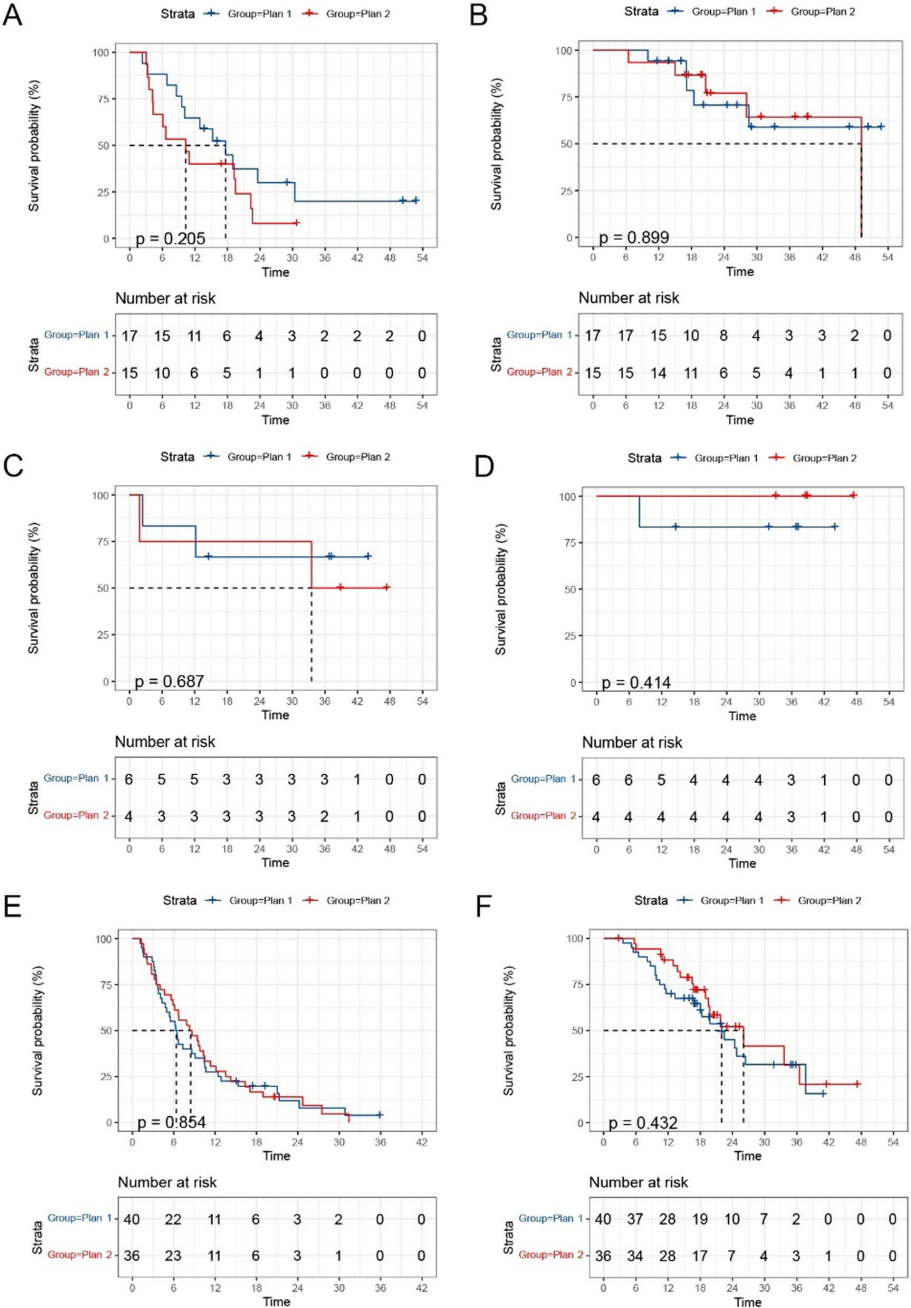
PFS curve **A** and OS curve **B** of 32 astrocytoma patients, PFS curve **C** and OS curve **D** of 10 oligodendroglioma patients and PFS curve **E** and OS curve **F** of 76 GBM patients by different target volumes. *PFS* Progression-free survival, *OS* Overall survival, *GBM* Glioblastomas

For GBM patients, the plan 1 subgroup showing a median PFS and OS were 6.3 and 22.0 months and the plan 2 subgroup showing a median PFS and OS of 8.3 and 26.1 months. For astrocytoma patients, median PFS were 17.67 and 10.3 months while median OS was not reached (NR) with plan 1 and plan 2 subgroups. For oligodendroglioma patients, median PFS were NR and 33.5 months while median OS was NR with plan 1 and plan 2 subgroups. There was no effect of treatment volume within either the GBM, astrocytoma or oligodendroglioma group (Fig. 3).

### Recurrence pattern

Of the 118 patients, a cohort of 90 patients occurred recurrence, in whom 13 patients were pathological confirmed to recur undergoing reoperation. The recurrence patterns were depicted in Table 3, mainly relapsed in the central (64.4%). We also observed that there were no differences in recurrence patterns among patients on different plans. Further investigation revealed that recurrence patterns had no effect on the median OS and PFS with central progression (21.8 months and 6.6 months), in-field progression (22.4 months and 4.6 months), marginal progression (33.9 months and 6.1 months), outside progression (37.6 months and 10.1 months), and subependymal (28.1 months and 10.3 months) (*p* = 0.480 and *p* = 0.598), respectively.

**Table 3:** Recurrence pattern and PFS in patients

Recurrence pattern	Plan1	Plan2	Astrocytoma (*N* = 21)	Oligodendroglioma(*N* = 3)	GBM (*N* = 66)	Plan1	Plan2	Plan1	Plan2
	*n* (%)			*n* (%)		mPFS (range)		Recurrence within 6 months (%)	
Central	31 (64.6)	27 (64.3)	11 (52.4)	2 (66.7)	45 (68.2)	5.5 (1.1–30.8)	6.7 (1.2–33.5)	51.6	40.7
In-field	4 (8.3)	6 (14.3)	3 (14.3)	0	7 (10.6)	9.1 (1.5–10.5)	4.7 (1.7–27.5)	25	50
Marginal	2 (4.1)	1 (2.3)	2 (9.5)	1 (33.3)	0	2.4 (2.4–30.4)	6.1	50	0
Distant	7 (14.6)	4 (9.5)	3 (14.3)	0	8 (12.1)	10.1 (5.7–24.2)	9.8 (8.3–31.4)	0	0
CSF-d	4 (8.3)	4 (9.5)	2 (9.5)	0	6 (9.1)	5.0 (3.7–18.9)	10.9 (2.3–24.7)	50	0

*CSF-d* Cerebrospinal fluid dissemination, *mPFS* Median progression-free survival, *GBM* Glioblastomas

For clarity of the role of peritumoral edema in tumor recurrence, we counted patients who relapsed in the edema region outside T1 + 2 cm which differed in whether it was irradiated or not in plan 1 and plan 2. There was no tumor completely recurred in this region. We found that plan 2 had slightly higher recurrence rate in this area than plan 1 (5/48 (10.4%) vs 6/42 (14.3%)), with no statistically significant effect (*p* = 0.576).

**Fig. 4:**
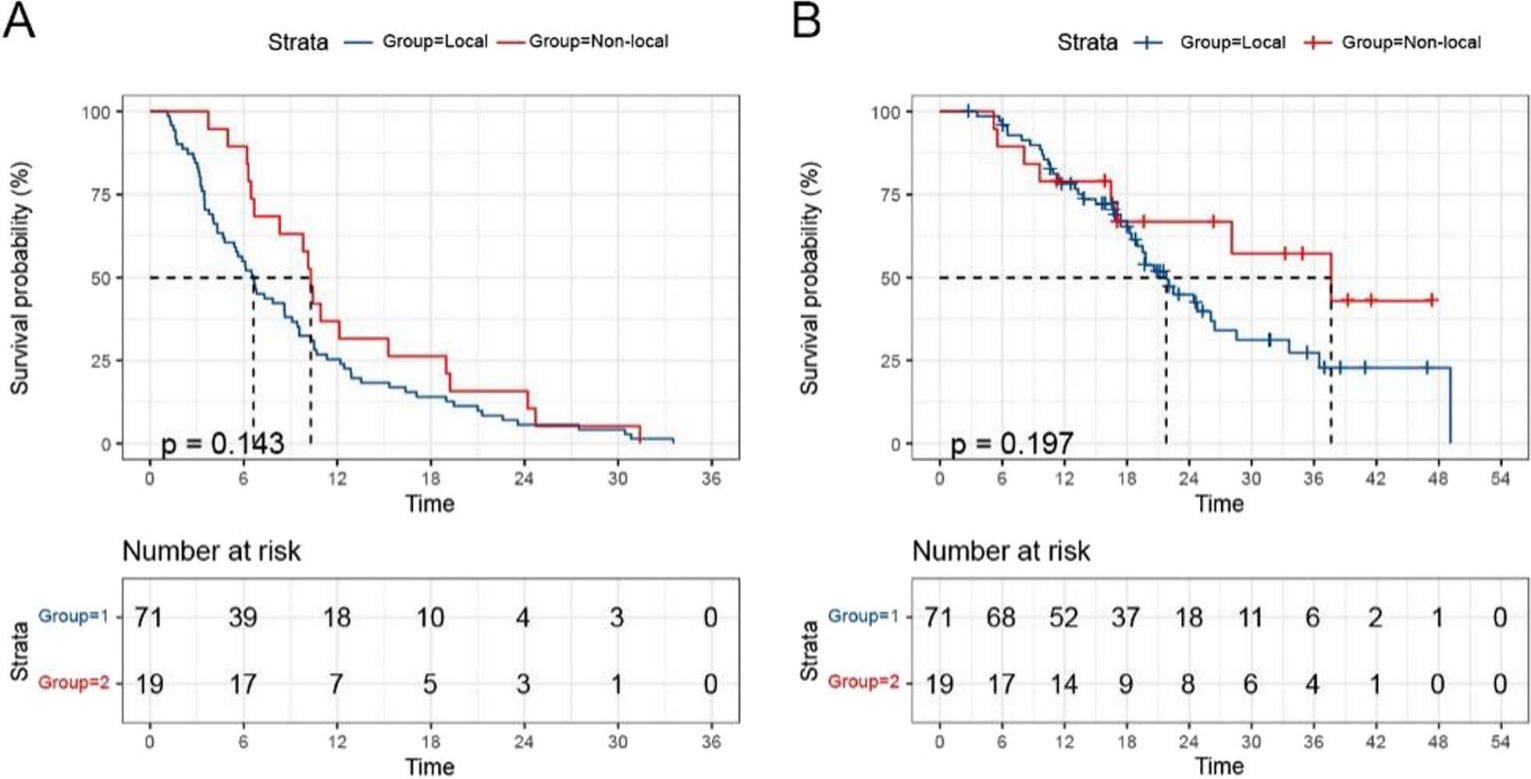
PFS and OS comparisons among different recurrence patterns of patients **A**, **B** *OS* Overall survival; *PFS* Progression-free survival

The recurrence patterns with central, in-field, and marginal were reclassified into local recurrence, whereas distant and CSF-d were considered the non-local recurrence. When we integrated the recurrence patterns into two groups, we found that patients with oligodendroglioma all relapsed in the local. And there was also no difference in PFS and OS between the two groups (*p* = 0.143; *p* = 0.197) (Fig. 4A–B).

### Risk factors related to recurrence patterns

**Fig. 5:**
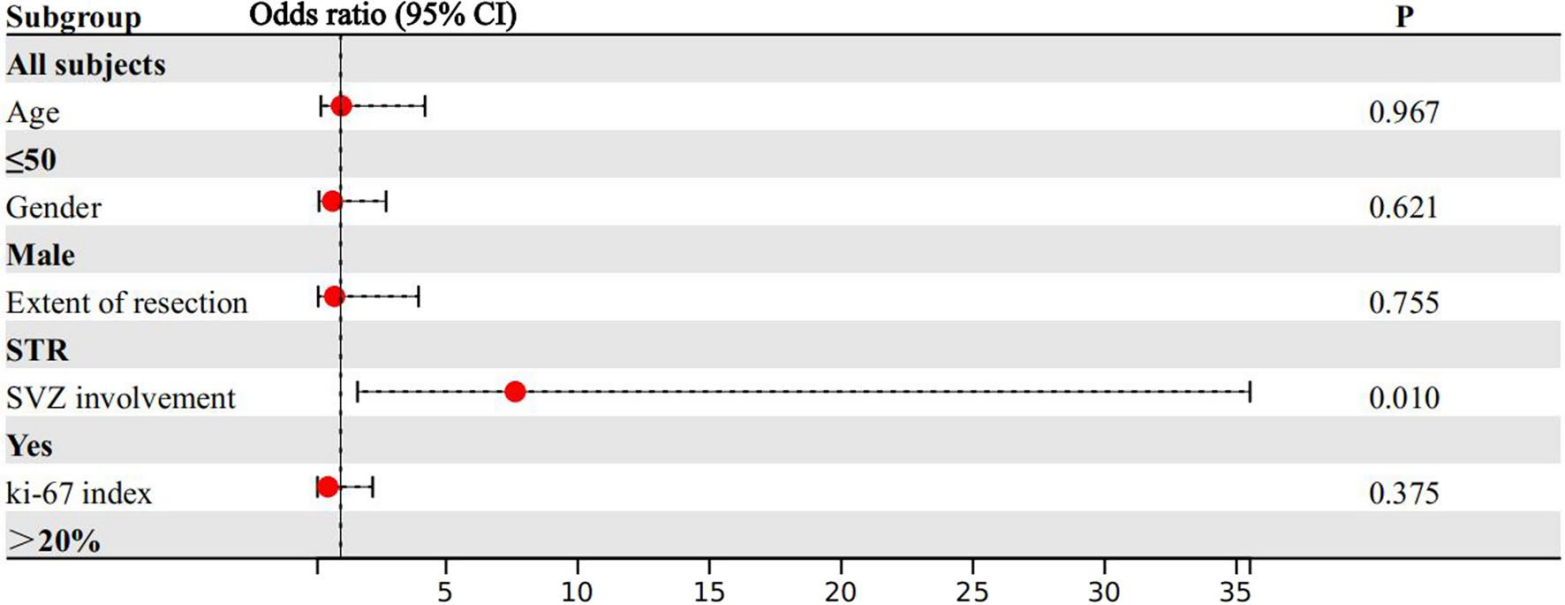
Forest plot of multivariate logistic regression analysis. Subgroups with a odds ratio larger than 1 were considered as risk factors, while those with a odds ratio less than 1 were considered as protective factors

To further identify the factors which contribute to different recurrences in patients with GBM, we performed a univariate analysis, as shown in Table 4. The frequency of SVZ involvement in the local group was significantly lower than that in the non-local group (36.5 vs 78.6%, *p* = 0.006). Concerning the molecular data, TERT promoter mutation was higher than that in the local group compared with the non-local group (85.7% vs 42.9%, *p* = 0.043). Although data on MGMT and TERT were reported related to recurrence patterns previously, they had missed so many values that it was excluded from the analysis in our study. SVZ involvement, gender, age, EOR, and Ki-67 index, which may be correlated with the recurrence pattern of GBM, were included in the multivariate analysis. In multivariate analysis (Fig. 5), SVZ involvement was the only independent risk factor for non-local recurrence (odds ratio = 7.629, *p* = 0.010).

**Table 4:** Comparison of baseline characteristics between different recurrence patterns in patients with GBM

Characteristics	Local(*N* = 52)	Non-local(*N* = 14)	*p*-value
Age(years), *n* (%)			0.491
≤ 50	12 (23.1)	5 (35.7)	
Gender, *n* (%)			0.745
Female	15 (28.8)	5 (35.7)	
BMI, *n* (%)			0.763
≤ 24	22 (42.3)	7 (50.0)	
Extent of resection, *n* (%)			0.708
GTR	43 (82.7)	11 (78.6)	
Laterality, *n* (%)			0.812
Left	26 (50)	9 (64.3)	
Right	21 (40.4)	4 (28.6)	
Bilateral	5 (9.6)	1 (7.1)	
SVZ involvement, n (%)			**0.006**
Yes	19 (36.5)	11 (78.6)	
Cerebral midline involvement, *n* (%)			1.000
Yes	31 (59.6)	8 (57.1)	
Brain cortex involvement, *n* (%)			0.753
Yes	16 (30.8)	5 (35.7)	
MGMT promoter, *n* (%)			0.686
Methylated	11 (44.0)	4 (57.1)	
TERT promoter, *n* (%)			**0.043**
Mutated	18 (85.7)	3 (42.9)	
Ki-67 index, *n* (%)			0.753
≤ 20	16 (30.8)	5 (35.7)	
ATRX, *n* (%)			1.000
Mutated	36 (87.8)	11 (91.7)	
BRAF, *n* (%)			1.000
Mutated	3 (7.7)	1 (9.1)	
P53 expression, *n* (%)			1.000
Mutated	34 (75.6)	8 (72.7)	
CD34, *n* (%)			0.762
Mutated	27 (60.0)	7 (53.8)	
Time of radiation, *n* (%)			1.000
≤ 42d	30 (57.7)	8 (57.1)	

*GTR* Gross total resection, *STR* Subtotal resection, *GBM* Glioblastomas, *MGMT* O6-methylguanine-DNA-methyltransferase gene, *TERT* Telomerase reverse transcriptase, *SVZ* Subventricular zone, *BRAF* v-raf murine sarcoma viral oncogene homolog B1, *p53* Protein 53, *CD34* Hematopoietic progenitor cell antigen CD34; In bold: *p* value less than 0.05

### Toxicity

Pseudoprogression/RNs occurred in 21 patients out of 118 patients (17.8%). There were 17 patients with pseudoprogression/RNs among patients with failure (*n* = 90, 18.9%). The incidence of pseudoprogression/RN was not substantially higher after treatment including peritumoral edema (20.6% vs. 14.5%, *p* = 0.388).

## Discussion

Adult-type diffuse gliomas are infiltrative and the extent of infiltration is the most important criterion for defining CTV. Because of the disparity between actual tumor existence and that estimated by CT or MRI, delineation of CTV for untreated or resected glioma remains a contentious and difficult issue. To date, many RT clinical trials were performing by varying fractionation, dose, and target region to extend the OS and PFS, yet none resulted in significant changes to outcomes or recurrence patterns [21, 23, 24]. Adult-type diffuse gliomas still typically use 2–3 cm expansion margins as outlined in RTOG or EORTC protocols. Both of them assigned T1-weighted MRI enhancement area and resection cavity into GTV while RTOG encompasses additional T2/Flair hyperintensity region (peritumoral edema area). RTOG protocol, developed by autopsy series [25] and MRI-guided stereotactic biopsies [26], has remained relatively unchanged over the past two decades. However, in many early comparisons of whether target contained edema or not, the inclusion of edema did not affect the pattern of tumor recurrence but rather increased the risk of radiation injury [16, 27]. Nie et al. found microscopic tumor extension (ME) value was 1.29 ± 0.54 cm for adult-type diffuse gliomas based on histologic sections and recommend 1.50 cm and 2.00 cm CTV margins [9]. Based on these findings, the EORTC protocol was developed. However, these results contrast with some previous studies [14, 28], who found that smaller boost fields increased marginal tumor recurrences. The inconsistent results of these studies may be related to RT techniques at different times or institutions and differed ways of comparing edema studies in various studies. This work establishes a clear direction to follow to better address this issue. In our institution, the target definitions are mainly clustered into two main groups which were designed under the RTOG and EORTC protocols. The main difference is whether the 60 Gy IDL includes the preoperative and postoperative edema areas. First, we compared the outcomes between the two groups and the results showed no significant difference in PFS and OS, which is consistent with current findings. It is well established that expanding the treatment margin can significantly increase the amount of brain tissue exposed to radiation [16]. Compared with plan 2, pseudoprogression/ RNs are more common in plan1 (20.6% vs 14.5%), but they did not reach statistical significance. The trend in RT has been to decrease treatment margins. Therefore, a small RT field may be more appropriate for patients with small edema regions and poor health.

In our study, the target deliberately involved edema made no difference in recurrence patterns and the most common recurrence occurred in the central field (64.4%), which was a little lower than previous research [8, 27, 29, 30]. There was no significant difference in recurrence outside T1 + 2 cm area between the two target delineation methods, suggesting that the inclusion of edema beyond 2 cm in RT did not significantly reduce relapsed rate at this site. We subsequently classified those with above 20% of the recurrence volume overlaps within 60 Gy IDL volume as local recurrence while the rest as non-local recurrence. Under this classification, we found the recurrence patterns of patients with oligodendroglioma were all local and there were no significant differences in prognosis. Moreover, for non-local recurrence, we identified SVZ involvement and TERT promoter wild-type were risk factors among the patients with GBM. TERT, a rate-limiting component of the telomerase holoenzyme, induces telomere maintenance [31, 32]. Previous studies revealed the role of TERT in the classification of molecular subtypes [33, 34] as well as demonstrated that patients with TERT promoter mutation had relatively poor outcomes [35–37]. However, the role of TERT in recurrence patterns hasn't been explicitly reported and additional larger studies will be required to confirm this association. After multivariate analysis, SVZ involvement was identified as an independent factor for non-local recurrence in our study. Adult-type diffuse gliomas are made up of multiple populations of tumor cells, among which those with the ability to initiate tumor development, herein referred to as glioma stem cells (GSCs), are of utmost importance. There is increasing evidence that GSCs contribute to tumor recurrences [38, 39]. In fact, GSCs can migrate out of the tumor mass and reach the SVZ, a neurogenic niche persisting after birth. Once nested in the SVZ, GSCs can escape a surgical intervention and resist treatments [40]. Numerous clinical studies have implicated the closer to the SVZ induced decreased survival and distant relapse [41, 42]. Regrettably, we did not identify the significance of recurrence patterns in MGMT, gender, and EOR which had been reported in other studies [43]. These variables are easily acquired from patients and used to predict the different patterns of relapse in patients with adult-type diffuse gliomas.

We have to mention that there are several limitations of this study. One is selection bias because it’s a retrospective study with a relatively small sample size of a single center. The other one is that the determination of recurrence is difficult and to some degree subjective. Finally, the detecting of biomarkers didn’t perform individually, so the data dearth may impact the data analysis.

## Conclusion

Delineating the area of peritumoral edema doesn’t influence the prognosis and recurrence patterns for patients with adult-type diffuse gliomas. We also observed that patterns of failure differed according to clinical and molecular characteristics. Therefore, the delineation of the target area should refer to the size of peritumoral edema and characteristics of the tumor. For patients with SVZ involvement, TERT promoter wild-type, and good health, we recommend including all areas of edema in CTV. A smaller target area may be appropriate if the patient is in poor condition or has substantial peritumoral edema.

## Data Availability

The datasets used and/or analyzed during the current study are available from the corresponding author on reasonable request.

## Abbreviations

GTR: Gross total resection
STR: Subtotal resection
GBM: Glioblastomas
SVZ: Subventricular zone
CSF-d: Cerebrospinal fluid dissemination
IDH: Isocitrate dehydrogenase
TERT: Telomerase reverse transcriptase
MGMT: O6-methylguanine-DNA methyltransferase promoter
1p/19q: Chromosome arms 1p and 19q
BRAF: V-raf murine sarcoma viral oncogene homolog B1
p53: Protein 53
CD34: Hematopoietic progenitor cell antigen CD34
OS: Overall survival
PFS: Progression-free survival
